# Effectiveness and Safety of Lidocaine-Nifedipine Combination in Patients With Acute and Chronic Anal Fissure: A Retrospective, Multi-centric, Post-marketing Surveillance Study

**DOI:** 10.7759/cureus.103680

**Published:** 2026-02-15

**Authors:** G Santhi Vardhani, R S Arun, Paramjeet Singh Sandhu, Rajan Madan, M Shenthil Prabhu, Snehal Purandare, Tarun Agarwal, Anjana Yadav, Sanjay Kamble

**Affiliations:** 1 Department of General Surgery, Yashoda Hospital, Secunderabad, IND; 2 Department of Gastroenterology, The Madras Medical Mission Hospital, Chennai, IND; 3 Department of Surgery, Dr. Sandhu's Piles Clinic, Amritsar, IND; 4 Department of Surgery, BLK-Max Super Speciality Hospital, New Delhi, IND; 5 Department of Surgical Gastroenterology, Tiruppur Gastro and Laparoscopic Centre, Tiruppur, IND; 6 Department of General Surgery, Navale Hospital, Pune, IND; 7 Department of Surgery, Gopi Krishna Laser Stone Centre, Lucknow, IND; 8 Medical Affairs, Samarth Life Sciences Pvt Ltd, Mumbai, IND

**Keywords:** acute, anal fissure, calcium channel blockers, chronic, lidocaine, nifedipine

## Abstract

Background

Anal fissures (AFs) are common and painful conditions that are frequently managed surgically; however, they are limited by fecal incontinence. Pharmacological management with calcium channel blockers has recently gained acceptance. This study aimed to evaluate the effectiveness and safety of twice-daily nifedipine (0.3% w/w) and lidocaine (1.5% w/w) cream in patients with acute AF (AAF) and chronic AF (CAF).

Methods

This retrospective, multi-centric, post-marketing surveillance study was conducted over a period of four weeks in the Department of Gastroenterology and Surgery across 46 institutes. The study included data of 431 patients with AAF (n=290) and CAF (n=141). The primary outcome measure was the percentage of patients with healed AFs, assessed at Weeks 1, 4, and 8. The secondary outcome measures were pain relief, evaluated using a 5-point VAS; percentage of patients with bleeding per anus; and adverse events (AEs), assessed at baseline and weekly follow-up intervals for eight weeks.

Results

At eight weeks, AF resolved significantly compared to the baseline (p<0.0001). During similar intervals, the incidence of bleeding per anus decreased significantly (p<0.0001), coupled with a significant reduction in the VAS score (p<0.0001). Though the resolution of AFs was comparable between AAF and CAF (p>0.05), the patients with AAF had a significantly greater reduction in bleeding per anus at Weeks 7 and 8 (p<0.05), with a significant decrease in the VAS score at Week 8 (p<0.05). All reported AEs were mild and self-limiting.

Conclusion

Topical nifedipine-lidocaine resulted in significant AF resolution, with significant pain relief and reduction in bleeding per anus, together with excellent tolerability.

## Introduction

Anal fissures (AFs), among the most painful anal conditions, significantly affect quality of life and present as a longitudinal or elliptical tear in the anoderm, distal to the dentate line and proximal to the anal verge [1]. In proctology consultations, the estimated prevalence of AFs is about 10% [2], with approximately 342,000 new cases diagnosed annually in the United States [3], while in India, AFs have a prevalence of around 18% among patients with anorectal disorders [4].

The pathophysiology of AFs is complex and involves anal sphincter hypertonicity and compromised blood flow, hindering the healing process [5]. Surgical options, including lateral internal sphincterotomy, are traditionally employed to prevent fissure recurrence; however, these interventions carry risks, including persistent fecal incontinence [6]. Recently, non-invasive pharmacological therapies focusing on sphincter pressure reduction and healing have gained traction. Agents such as glyceryl trinitrate, calcium channel blockers, and botulinum toxin have been prominently studied [7], though treatment responses vary owing to differing methodologies and follow-up periods across studies [8].

Topical agents such as nifedipine, a calcium channel blocker, have demonstrated effectiveness in reducing pain and promoting healing by lowering anal resting pressure and improving local perfusion [8]. Studies suggest that combining nifedipine with lidocaine may enhance treatment outcomes by simultaneously alleviating pain and facilitating healing [1,5]. However, blood pressure-lowering effects of nifedipine are similar to those of nitroglycerin ointment, though headaches are less frequent, affecting around 25% patients [8]. While studies support the effectiveness of this combination for healing and symptomatic pain relief, data on its impact on bleeding fissures and safety in the Indian population remain limited [9,10]. The effectiveness of this combination has been tried either in patients with acute AF (AAF) or chronic AF (CAF) [9-11], and seldom in a mixed population with AFs [12]. Moreover, these studies had a small sample size. Thus, this study aimed to assess the safety and effectiveness of a fixed-dose combination of nifedipine-lidocaine primarily in promoting healing and secondarily in relieving pain and reducing bleeding in patients with AAF and CAF.

## Materials and methods

Study design and ethics

This retrospective, multi-centric, post-marketing surveillance study was conducted over a period of four weeks in the Department of Gastroenterology and Surgery of 46 institutes across India. The study was approved by the Institutional Ethical Committee. Due to its retrospective study design, written informed consent of the patients was waived off.

Population

This study involved a review of electronic case records of patients with AF who were treated with the topical cream containing nifedipine-lidocaine. The data of male and female patients aged over 18 years and diagnosed with AAF and CAF, present for <6 and >6 weeks, respectively, [2] were included.

However, patients with missing data, a recorded history of recurrent anal fissures, anorectal malformations, and a history of anal surgery, or associated conditions such as severe anemia, cancer, fistula, abscess, Crohn’s disease, HIV-related anal ulcer, tuberculosis ulcer, leukemic ulcer, pregnancy, third- and fourth-degree hemorrhoids, diabetes mellitus, or hypertension were excluded. Additionally, those who received oral or topical therapy for AF or had a history of chronic constipation were excluded.

Data collection

The study included data from 431 patients suffering from AAF (n = 290) and CAF (n = 141). The patients with complete records of baseline and follow-up intervals were included in the analysis.

Study treatment

The study involved patients who applied the cream twice daily for at least eight weeks. All the patients were adults and received the same dosage. The composition of the cream was nifedipine IP 0.3% w/w and lidocaine IP 1.5% w/w. As instructed by the treating surgeons, all the patients followed the standard procedure, including lying down on the left side with the left leg straight, the right leg bent with the knee in the air, and with the help of a gloved finger applying approximately 3 g of cream topically on the peri-anal area 1-1.5 cm inside the anal canal from the anoderm.

In addition to the topical cream, standard of care included a high-fiber diet, a 10-15 minute warm sitz bath three times daily, and increased water intake. For AF-associated severe pain, tablet Acetaminophen (500 mg) was used, as needed.

Follow-up

From the available records, the treating surgeons recorded the data of selected patients at baseline, including clinical examination findings related to AF characteristics and bleeding per anus. They also noted the baseline pain score, based on a 5-point visual analog scale (VAS) ranging from 0 (no pain) to 4 (worst pain).

Data from weekly follow-up intervals at Weeks 1, 2, 3, 4, 5, 6, 7, and 8 were obtained from the case records of the patients. Safety and effectiveness data were recorded at each follow-up interval except Weeks 4 and 8, when clinical examination data were available for AF healing. Additionally, at each follow-up interval, the treating surgeons recorded pain relief, bleeding per anus, and adverse events (AEs).

Study outcomes

The primary effectiveness outcome was the percentage of patients with completely healed AFs, defined as complete epithelialization of the mucosa at the fissure area, based on clinical examination data. Other outcome measures included pain relief, based on the 5-point VAS at baseline, and the percentage of patients with bleeding per anus, both recorded at each follow-up interval.

Safety evaluation included available AE records, either reported spontaneously by the patients or based on voluntary inquiry during follow-ups.

Sample size estimation

The data of 431 patients with AFs were included from 46 institutes, with 8-10 patients from each site. The patients with complete medical records at each follow-up interval were included, and data were analyzed for effectiveness and safety.

Statistical analyses

Statistical analyses were performed with R-4.4.3 for Windows. The categorical and continuous variables were represented as frequency (percentage) and mean ± standard deviation (SD), respectively. The chi-square goodness of fit test, followed by the pairwise proportional test and repeated measures ANOVA, followed by Bonferroni’s multiple comparison test, were used to assess changes in categorical and continuous variables over the study period, respectively. Subgroup analysis was performed with the chi-square test and independent sample t-test for categorical and continuous variables, respectively. A two-tailed probability (p) value of < 0.05 was considered statistically significant.

## Results

The patients were predominantly male (n = 272, 63.11%), with a mean age of 42.03 ± 13.98 years. The mean BMI was 24.05 ± 3.82 kg/m². Regarding adverse habits, 92 (21.35%) patients smoked, 84 (19.49%) consumed alcohol, and 25 (5.80%) reported drug abuse. The majority of patients were diagnosed with AAF (n = 290, 67.29%), while 141 (32.71%) had CAF. Bleeding per anus was present in 75.87% (n = 327) of patients. The mean baseline VAS score for pain was 2.53 ± 0.96 (Table 1).

**Table 1:** Demographic and baseline characteristics BMI: Body mass index; SD: Standard deviation; VAS: Visual analog scale

Characteristics	n = 431
Age, years, mean ± SD	42.03 ± 13.98
Sex, n (%)	
Female	159 (36.89)
Male	272 (63.11)
BMI, kg/m^2^, mean ± SD	24.05 ± 3.82
Adverse habits, n (%)	
Smoking	92 (21.35)
Alcohol	84 (19.49)
Drug abuse	25 (5.80)
Diagnosis, n (%)	
Acute anal fissure	290 (67.29)
Chronic anal fissure	141 (32.71)
Bleeding per anus, n (%)	327 (75.87)
VAS score, mean ± SD	2.53 ± 0.96

Resolution of AF

At baseline, all patients had AFs (n = 431, 100%). Over the study period, the patients with AFs decreased sequentially from 42.23% (n = 182) at Week 4 to 3.94% (n = 17) at Week 8. At the end of the study, there was a significant resolution of AFs compared to the baseline (p < 0.0001). Post hoc pairwise analysis showed significant resolution across all intervals studied (p < 0.0001). The subgroup analysis revealed a significant resolution of AFs in patients with AAF and CAF (p < 0.0001). Though the reduction in the incidence of AFs was slightly greater in patients with AAF, both groups did not differ significantly at any of the intervals (p > 0.05) (Table 2).

**Table 2:** Primary outcome: resolution of anal fissure AAF: Acute anal fissure; CAF: Chronic anal fissure; *Chi-square test for between-group analysis (AAF vs CAF); DF: Degrees of freedom; #Goodness of fit Chi-square test for within-group analysis

Intervals	Total (n=431)	AAF (n=290)	CAF (n=141)	DF	Effect size	Χ^2^ value	p*
Baseline	431 (100.00%)	290 (100.00%)	141 (100.00%)				-
Week 4	182 (42.23%)	127 (43.79%)	55 (39.01%)	1	0.0719	2.23	0.1358
Week 8	17 (3.94%)	9 (3.10%)	8 (5.67%)	1	0.0655	1.85	0.1738
p^#^	< 0.0001	< 0.0001	< 0.0001				

Bleeding per anus

At baseline, 327 (75.87%) patients had bleeding per anus. Similar to the AF resolution, the proportion of patients with bleeding per anus decreased sequentially from 288 (66.82%) at Week 1 to 9 (2.09%) at Week 8. At Week 8, this decrease in the proportion of patients with bleeding per anus was statistically significant compared to the baseline (p < 0.0001). Post hoc pairwise analysis revealed a significant decrease in the incidence of bleeding per anus at all the intervals except between baseline and Week 1 (p = 0.151), Weeks 3 and 4 (p = 0.243), Weeks 4 and 5 (p = 0.571), Weeks 5 and 6 (p = 1.000), Weeks 5 and 7 (p = 0.095), Weeks 6 and 7 (p = 1.000), Weeks 6 and 8 (p = 1.000), and Weeks 7 and 8 (p = 1.000). Moreover, at baseline, both AAF and CAF patients had comparable bleeding per anus (p = 0.806). Throughout the study period, the incidence of bleeding per anus declined progressively and significantly in both the groups (p < 0.0001), it was lower in patients with AAF than those with CAF; however, it reached statistical significance only at Weeks 7 (p = 0.027) and 8 (p = 0.004), thereby suggesting that cream provides significantly greater reduction in incidence bleeding per anus in AAF (Table 3).

**Table 3:** Secondary outcome: bleeding per anus AAF: Acute anal fissure; CAF: Chronic anal fissure; *Chi-square test for between-group analysis (AAF vs CAF); DF: Degrees of freedom; #Goodness of fit Chi-square test for within-group analysis

Intervals	Total (n=431)	AAF (n=290)	CAF (n=141)	DF	Effect size	Χ^2^ value	p*
Baseline	327 (75.87%)	219 (75.52%)	108 (76.6%)	1	0.0118	0.06	0.806
Week 1	288 (66.82%)	191 (65.86%)	97 (68.79%)	1	0.0293	0.37	0.544
Week 2	185 (42.92%)	119 (41.03%)	66 (46.81%)	1	0.0547	1.29	0.256
Week 3	83 (19.26%)	54 (18.62%)	29 (20.57%)	1	0.0231	0.23	0.631
Week 4	53 (12.30%)	32 (11.03%)	21 (14.89%)	1	0.0551	1.31	0.252
Week 5	31 (7.19%)	19 (6.55%)	12 (8.51%)	1	0.0357	0.55	0.460
Week 6	19 (4.41%)	11 (3.79%)	8 (5.67%)	1	0.0431	0.80	0.372
Week 7	11 (2.55%)	4 (1.38%)	7 (4.96%)	1	0.1066	4.90	0.027
Week 8	9 (2.09%)	2 (0.69%)	7 (4.96%)	1	0.1403	8.48	0.004
p^#^	< 0.0001	< 0.0001	< 0.0001				

Pain relief

At baseline, the mean VAS score was 2.53 ± 0.96. With the application of cream, there was a significant pain relief (p < 0.0001), with the VAS score decreasing from 2.30 ± 1.07 at Week 1 to 0.11 ± 0.44 (0, 0) at Week 8. Post hoc pairwise analysis demonstrated a significant reduction in the VAS score across all intervals (p < 0.0001), except between Weeks 6 and 7, Weeks 6 and 8, and Weeks 7 and 8 (all p > 0.05). A subgroup analysis revealed that the mean baseline VAS score was slightly greater in patients with AAF than those with CAF; however, the difference was not statistically significant (p = 0.052). Throughout the study period, the mean VAS score declined progressively and significantly in both the groups (p < 0.0001). Both the groups had comparable pain relief, except at Week 8, where the VAS score was significantly lower in the AAF group (p = 0.031) (Table 4).

**Table 4:** Secondary outcome: VAS score AAF: Acute anal fissure; CAF: Chronic anal fissure; DF: Degrees of freedom; *Independent sample t-test for between-group analysis (AAF vs CAF); #Repeated measures ANOVA followed by Bonferroni’s multiple comparison test for within-group analysis

Intervals	Total (n=431)	AAF (n=290)	CAF (n=141)	DF	t-test value	p*
Baseline	2.53 ± 0.96	2.59 ± 1.01	2.41 ± 0.84	429	1.95	0.052
Week 1	2.30 ± 1.07	2.34 ± 1.11	2.22 ± 0.99	429	1.13	0.258
Week 2	1.65 ± 1.04	1.66 ± 1.04	1.65 ± 1.03	429	0.09	0.925
Week 3	1.13 ± 0.97	1.11 ± 0.99	1.16 ± 0.94	429	0.51	0.611
Week 4	0.71 ± 0.92	0.68 ± 0.91	0.76 ± 0.94	429	0.84	0.403
Week 5	0.45 ± 0.77	0.43 ± 0.75	0.49 ± 0.82	429	0.73	0.465
Week 6	0.25 ± 0.57	0.22 ± 0.49	0.33 ± 0.70	429	1.68	0.095
Week 7	0.18 ± 0.51	0.16 ± 0.48	0.21 ± 0.58	429	0.89	0.376
Week 8	0.11 ± 0.44	0.08 ± 0.38	0.18 ± 0.55	429	1.95	0.031
p^#^	< 0.0001	< 0.0001	< 0.0001			

Safety

The incidence of AEs decreased progressively over time, from 14 (3.25%) at Week 1 to 0 (0.00%) at Week 8. At Weeks 1, 2, 3, and 4, the most frequently reported AE was burning sensation (8 (1.86%), 9 (2.09%), 8 (1.86%), and 3 (0.70%), respectively), while itching was the most commonly reported AE at Weeks 5 (n = 2, 0.46%) and 6 (n = 1, 0.23%). All the AEs were mild and resolved spontaneously, with none requiring discontinuation of the study drug (Table 5).

**Table 5:** Comparison of adverse event parameters in the follow-up period

Adverse events, n (%)	Follow-up intervals (n=431)							
	Week 1	Week 2	Week 3	Week 4	Week 5	Week 6	Week 7	Week 8
Watery discharge	2 (0.46%)	2 (0.46%)	3 (0.70%)	0 (0.00%)	0 (0.00%)	0 (0.00%)	0 (0.00%)	0 (0.00%)
Burning sensation	8 (1.86%)	9 (2.09%)	8 (1.86%)	3 (0.70%)	0 (0.00%)	0 (0.00%)	0 (0.00%)	0 (0.00%)
Headache	1 (0.23%)	0 (0.00%)	0 (0.00%)	0 (0.00%)	0 (0.00%)	0 (0.00%)	0 (0.00%)	0 (0.00%)
Itching	2 (0.46%)	2 (0.46%)	1 (0.23%)	1 (0.23%)	2 (0.46%)	1 (0.23%)	0 (0.00%)	0 (0.00%)
Slight dizziness	1 (0.23%)	0 (0.00%)	0 (0.00%)	0 (0.00%)	0 (0.00%)	0 (0.00%)	0 (0.00%)	0 (0.00%)
Total	14 (3.25%)	13 (3.02%)	12 (2.78%)	4 (0.93%)	2 (0.46%)	1 (0.23%)	0 (0.00%)	0 (0.00%)

## Discussion

The principal findings of the study demonstrated significant resolution of AFs, a decrease in bleeding per anus, and pain relief. The improvement in all the primary and secondary outcome measures was greater in patients with AAF compared to those with CAF; however, it reached statistical significance only for bleeding per anus and pain relief, and not for resolution of AFs. Additionally, the combination of nifedipine-lidocaine was well tolerated, with all the patients reporting spontaneously resolving mild AEs.

The present study demonstrated a significant reduction in the incidence of AFs over the study period, indicating substantial clinical improvement. The combination of nifedipine-lidocaine likely aids AF resolution through the analgesic properties of lidocaine and ability of nifedipine to decrease resting anal pressure. Nifedipine, known for its vasodilatory effects, enhances blood flow to the affected area, thereby alleviating ischemia and promoting healing. Its ability to reduce internal anal sphincter pressure addresses hypertonicity, which contributes to pain and delays healing in chronic lesions, creating a more favorable environment for regeneration [13]. Local anesthetics like lidocaine provide immediate pain relief by blocking nerve transmission, which is particularly beneficial during defecation, an activity that exacerbates pain in patients with AF [14]. By reducing pain perception, lidocaine encourages regular bowel habits, further supporting the healing process. In the present study, AF resolution was observed in 414 (96.06%) patients with AF, including 281 (96.1%) patients with AAF and 133 (94.33%) patients with CAF. Similarly, Albayati et al. reported that the healing rate was 90% (36/40) in patients with AAF and 83.33% (50/60) in those with CAF after eight weeks of topical treatment with nifedipine-lidocaine [12]. However, other studies by Perrotti et al. [5] and Momayez Sanat et al. [11] reported a remission rate of 94.5% (52/55) and 77.4% (41/53) in patients with CAF and AAF following treatment with topical nifedipine over six and eight weeks, respectively. Moreover, vasodilatory properties of nifedipine counteract the reduced blood supply typically seen in CAF, which allows for better healing of the mucosal surface and ultimately leads to decreased bleeding incidents over time [12]. At the end of the study, we observed that 422 (97.91%) patients did not have bleeding per anus, and similar findings were noted in 288 (99.31%) and 134 (95.04%) patients with AAF and CAF, respectively. Similarly, Kujur et al. reported that none of the patients with CAF had bleeding following eight weeks of treatment with 0.3% nifedipine [10].

The reduction in VAS scores likely reflects both fissure healing and pain relief, consistent with studies supporting the efficacy of topical agents. Overall, 406 (94.19%) patients with AFs had complete pain relief after eight weeks of treatment, while 279 (96.21%) and 127 (90.07%) patients with AAF and CAF, respectively, reported no pain. Moreover, all the patients with AFs, irrespective of AF type, had a significant reduction in the VAS score. Perrotti et al. reported significant pain reduction in the VAS score, with 87.3% (48/55) of nifedipine-treated patients experiencing complete pain relief compared to 10.9% (6/55) in the control group, while persistent pain remained in 30.9% (17/55) of controls [5]. Similar findings had been observed by Kujur et al. [10]. In another study, Perrotti et al. demonstrated that a combination of nifedipine-lidocaine led to a significant reduction in post-hemorrhoidectomy pain [15], thus illustrating analgesic properties in diseases of the anal canal, irrespective of underlying pathologies. The present study demonstrated a decline in the incidence of both AAF and CAF; however, AAF resolved to a slightly greater extent. This distinction highlights differences in pathophysiology and healing potential. AAF, typically present for less than six weeks, responds well to conservative treatment due to intact local blood supply and minimal sphincter hypertonicity. Pain in AAF primarily results from mechanical trauma during defecation, making topical anesthetics and vasodilators effective in symptom relief and healing [16]. Conversely, CAF, persisting for more than six weeks, often involves pathological changes such as persistent sphincter hypertonicity and reduced perfusion, which can delay healing. Moreover, compromised vascularity in CAF prolongs symptoms, sometimes necessitating more intensive medical or surgical interventions [17]. Katsinelos et al. reported that 0.5% topical nifedipine ointment, acting as a chemical sphincterotomy agent, significantly enhances AAF healing and may prevent chronic progression [18]. Altomare et al. highlighted that pain relief in chronic cases is substantial but may not occur within the same timeframe as AAF, underscoring the need for tailored treatment approaches [19].

The patients with AAF and CAF demonstrated significant reductions in bleeding per anus throughout the treatment period, but the reduction was more swift in patients with AAF than those with CAF, achieving statistical significance by Weeks 7 and 8. The variation in bleeding incidence between AAF and CAF can be attributed to differences in pathophysiology, healing dynamics, and treatment responses. In AAF, bleeding is often limited and results from recent trauma, such as passage of hard stools, causing superficial anodermal tears that heal quickly with appropriate management. Topical agents like nifedipine and lidocaine provide immediate pain relief and reduce anal sphincter spasm, facilitating smoother bowel movements while minimizing further trauma to the fissure. Improved local blood flow further accelerates healing, leading to a rapid decline in bleeding incidence, as observed in the present study and corroborated by previous research. For instance, Katsinelos et al. reported that AAF responds effectively to topical nifedipine, significantly reducing bleeding rates during defecation [18]. In contrast, CAF exhibits a more complex healing process, characterized by persistent sphincter hypertonicity and reduced blood supply, which contributes to prolonged inflammation and bleeding [17]. The presence of fibrotic tissue and chronic structural changes exacerbates symptoms, making healing slower compared to acute cases. It is further reported that scarring and vascular alterations in CAF coupled with sustained inflammation, lead to recurrent bleeding episodes [20]. Although nifedipine and lidocaine improve symptoms in CAF, prolonged vascular and muscular dysfunction delays complete resolution. It is observed that patients with CAF experience more frequent bleeding episodes due to sustained inflammation and sphincter hypertonicity [12,21]. Similarly, the prevalence of rectal bleeding in CAF compared to AAF is attributed to the persistent nature of symptoms associated with chronic lesions [16].

The present study highlights slightly higher VAS scores among AAF patients than CAF patients. While both groups experienced comparable pain relief, AAF patients reported significantly lower VAS scores at study completion, suggesting a more favorable treatment response. AAF is characterized by sharp pain during bowel movements due to recent trauma and inflammation [22]. Despite slower healing, CAF patients achieved significant pain relief over time with appropriate treatment, suggesting that both presentations respond to interventions aimed at reducing sphincter pressure and inflammation [17]. To the best of our knowledge, none of the available studies have compared pain reduction in patients with AAF and CAF. However, Albayati et al. demonstrated slightly better resolution of AAF than CAF [12], thereby indirectly suggesting better pain reduction in AAF compared to CAF.

The topical preparation was well-tolerated, as all AEs were mild and resolved spontaneously. Burning sensation could be attributed to the pharmacological action of nifedipine-lidocaine, which targets anal muscle tension and enhances blood flow but may irritate the surrounding tissue [12]. By Week 8, the absence of reported AEs suggests that the treatment was well-tolerated, indicating a successful resolution of initial irritation and discomfort. Similarly, Albayati et al. noted that patients treated with nifedipine-lidocaine experienced mild, self-limiting AEs such as headache, itching, and palpitations, which resolved with continued treatment [12]. In contrast, Perrotti et al. reported no AEs following topical nifedipine and lidocaine use [5]. Most AEs reported in this study were transient, with infrequent occurrences of headache, further supporting the tolerability of topical preparation. Studies have documented lower AE rates with nifedipine compared to other topical agents such as diltiazem and glyceryl trinitrate, reinforcing its favorable safety profile for long-term AF management [8,23].

This study has a few limitations. First, as a retrospective analysis, it is prone to biases in data collection and lacks control over confounding variables, potentially affecting treatment outcomes. Second, reliance on patient-reported outcomes, such as VAS scores, introduces potential reporting biases. Third, the short follow-up period for safety assessment may overlook long-term AEs, which could provide further insights into treatment tolerability. Fourth, recurrence of AF could not be assessed, mainly due to lack of data. Finally, the retrospective design, while allowing for broader patient inclusion, limits the ability to establish definitive causal inferences regarding the effectiveness and safety of the nifedipine-lidocaine fixed-dose combination.

## Conclusions

Over the period of eight weeks, topical nifedipine-lidocaine led to significant resolution of AFs, associated with significant pain relief and reduction in bleeding per anus. The resolution of AFs was comparable between AAF and CAF; however, pain relief and reduction in bleeding per anus were significantly greater in AAF than in CAF. Moreover, topical nifedipine-lidocaine was safe and well-tolerated.
